# The Impact of Long-Term Physical Activity Interventions for Overweight/Obese Postmenopausal Women on Adiposity Indicators, Physical Capacity, and Mental Health Outcomes: A Systematic Review

**DOI:** 10.1155/2016/6169890

**Published:** 2016-05-16

**Authors:** Amanda Baker, Héloïse Sirois-Leclerc, Heather Tulloch

**Affiliations:** ^1^School of Psychology, University of Ottawa, 136 Jean-Jacques Lussier Street, Ottawa, ON, Canada K1N 6N5; ^2^Prevention and Rehabilitation Centre, University of Ottawa Heart Institute, 40 Ruskin Street, Ottawa, ON, Canada K1Y 4W7

## Abstract

Physical activity interventions have recently become a popular strategy to help postmenopausal women prevent and manage obesity. The current systematic review evaluates the efficacy of physical activity interventions among overweight and obese postmenopausal women and sheds light on the behavioral change techniques that were employed in order to direct future research.* Method*. Five electronic databases were searched to identify all prospective RCT studies that examine the impact of physical activity on adiposity indicators, physical capacity, and/or mental health outcomes among healthy, sedentary overweight, and obese postmenopausal women in North America. The behavior change technique taxonomy was used to identify the various strategies applied in the programs.* Results*. Five RCTs met the inclusion criteria. The findings showed that adiposity indicators and physical capacity outcomes significantly improved following long-term interventions; however, mental health outcomes showed nonsignificant changes. Furthermore, 17 behavior change techniques were identified with the taxonomy across all trials. The intrapersonal-level techniques were the most common.* Conclusion*. Physical activity interventions had a positive effect on adiposity measures and physical capacity. Future research should focus on testing the effectiveness of physical activity interventions on mental health and incorporate strategies at the individual and environmental level to maximize the health impact on the population.

## 1. Introduction

Overweight/obesity is one of the most widespread health problems on a global scale [1] and the most significant contributor to poor health, exceeding both undernutrition and infectious diseases [2]. The issue has become a primary concern for public health officials and practitioners as more than 50% of Canadians and Americans are classified as either obese or overweight [3, 4]. In recent years, the prevalence of obesity and overweight has remained stable except among middle aged and older women; trends have continued to rise for this group [3]. In North America, the prevalence of obesity is higher among middle aged women (40–59 years old), followed closely by 60-year-old and older women (39.5% and 35.4%, resp.) [5]. The North American Menopause Society [6] noted that by 2025 the number of postmenopausal women is projected to increase substantially, and those who are overweight and obese will presumably need additional clinical care and weight intervention. Evidently, this subpopulation requires attention in order to understand and prevent such prospective trends.

Various health-related consequences have been associated with excessive body weight. Overweight and obesity can have adverse effects on nearly every aspect of health from contributing to chronic conditions such as osteoarthritis [7] to interfering with sexual function [8], breathing [9], mood [10], and social interactions [11]. More specifically, Yatsuya et al. [12] found that the hazard ratio of stroke increased with higher levels of body mass index (BMI) among obese black and white women (1.10, 95% Confidence Interval (CI) (0.97, 1.24); 1.25, 95% CI (1.07, 1.46), resp.). Among older women, more severe vasomotor symptoms (e.g., night sweat and hot flashes) during the menopause transition and postmenopausal phase are experienced as BMI increases [6]. Overweight and obese postmenopausal women also experience increased risk for type II diabetes, breast cancer, coronary heart disease, and depression. For instance, a meta-analysis of 18 cohort studies [13] found that the relative risk of type II diabetes for obese men and women compared to normal weight individuals was 7.19 (95% CI (5.74, 9.00)) and for overweight men and women was 2.99 (95% CI (2.42, 3.72)). Based on a pooled analysis of 7 cohort studies [14], the relative risk of breast cancer among overweight postmenopausal women was 1.26 (95% CI (1.09, 1.46)). A longitudinal study by Li et al. [15] found that the relative risk of coronary heart disease was 3.44 (95% CI (2.81, 4.21)) for women who were obese (BMI ≥ 30 kg/m^2^) and sedentary (exercise < 1 hr/wk), 2.48 (95% CI (1.84, 3.34)) for women who were active but obese, and 1.48 (95% CI (1.24, 1.77)) for women who had a healthy weight but were sedentary compared to the normal BMI and physically active group. Even modest weight gain (8 to 20 pounds) during adulthood was associated with 27% (95% CI (12% to 45%)) increased risk of coronary heart disease compared to women with a stable weight after adjusting for physical activity and other cardiovascular risk factors. Based on a meta-analysis of longitudinal survey studies [16], the pooled odds ratio for the association between obesity at baseline (time 1) and increased depression at follow-up (time 2) was 1.55 (95% CI (1.22, 1.98)). The status of overweight at baseline also increased the risk of onset of depression at follow-up (odds ratio 1.27, 95% CI (1.07, 1.51)).

According to Rosano et al. [17], the major risks associated with being overweight tend to be experienced by those who drastically gain weight during midlife adulthood. Body weight in women tends to steadily increase from the late 20s to 60 years of age with the greatest increase in weight occurring shortly after menopause or during the 5th decade [18]. The increase in weight during the menopause stage and beyond is most harmful for women [19]. Therefore, many overweight sedentary postmenopausal women experience negative effects such as high blood pressure and the aforementioned conditions, which can elicit other serious health problems [20].

Engaging in regular moderate physical activity is strongly associated with better physical and psychological health outcomes and has been shown to reduce negative effects associated with overweight and obesity [21, 22]. For example, physical activity was found to reduce abdominal fat and total percentage of body fat even without changes in body weight [23]. A study by McCullough et al. [24] found that mild or intense physical activity may reduce the risk of breast cancer. Furthermore, a review by Paluska and Schwenk [25] reported that physical activity was associated with reduced depressive and acute anxiety symptoms.

Wen et al. [26] found that even small changes or adjustments in health behaviors can have an impact on overall health. Based on their cohort study, individuals in the low activity group who exercised for an average of 92 minutes per week (95% CI (71.0, 112.0)) or 15 minutes a day (SD = 1.8) had a 14% reduced risk of all-cause mortality (hazard ratio 0.86, 95% CI (0.81–0.91)) and a 3-year longer life expectancy compared to the inactive group. The benefits were found for all age groups (20–39, 40–59, and ≥60) and for both men and women. A meta-analysis conducted at the Harvard School of Public Health [27] found that as little as 150 minutes (2.5 hours) of physical activity a week can significantly reduce the risk of heart disease by approximately 14% among adult men and women. About 300 minutes (5 hours) a week reduced the risk of heart disease including heart attacks, angina, and bypass surgeries by 20% compared to adults who did not engage in physical activity. The study also found that physical activity had a significantly stronger effect in reducing the risk of heart disease in adult women (33%) compared to adult men (22%). The benefits associated with minimal behavior change in terms of physical activity are abundant, and such changes are deemed crucial in the prevention and treatment of obesity.

Sustained physical activity is necessary to achieve health benefits. According to Peterson and Ward-Smith [18], short-term benefits of physical activity among overweight and obese women are limited to less fatigue and increased cardiovascular fitness as opposed to weight loss, whereas the long-term effects of physical activity are weight loss and improved physical fitness to help overweight and obese women achieve an ideal weight and standard of living [18]. Some studies have also documented that long-term physical activity significantly improves psychological functioning [28]. A review by Folkins and Sime [29] found that long-term physical activity improved self-confidence and self-esteem in male and female children and adults. Adult men who were subjected to a long-term intervention demonstrated reduced mental stress [30] and reduced Type A behaviors (i.e., competitive and aggressive behaviors) [31]. However, consensus for the particular effects of long-term physical activity on postmenopausal populations is less clear because a synthesis of the research is yet to be provided in the literature. Evaluating the efficacy of long-term (≥6 months) physical activity interventions among both overweight and obese postmenopausal women is warranted.

The current systematic review will evaluate the efficacy of physical activity interventions on adiposity indicators, physical capacity measures, and mental health outcomes among sedentary overweight/obese postmenopausal women. Another goal of the review is to identify the various behavioral change techniques that were employed in the interventions to help inform future research and interventions that target this growing population.

## 2. Methods

### 2.1. Data Sources and Search Strategies

Five electronic databases, MEDLINE, Cochrane CENTRAL, PubMed, PsycINFO, and Web of Science, were searched for relevant studies from the past 10 years. Reference lists of all included studies were searched for further relevant studies. The search terms included obesity terms and physical activity terms as well as filters for randomized controlled trials (RCTs), human subjects, English articles, adult females, and being published between January 2005 and June 2015.

### 2.2. Inclusion Criteria

Articles were selected based on several inclusion criteria.

#### 2.2.1. Types of Studies

Published RCTs providing ≥6 months of follow-up data after randomization were included. Interventions that were less than 6 months were excluded because previous researches have used a 6-month cut-off when evaluating metabolic, biochemical, haematological, and muscular change from physical activity interventions [32, 33].

#### 2.2.2. Types of Participants

Studies were included if the sample is comprised of postmenopausal women living in Canada or the United States because the current levels of physical activity are very much alike. Based on Canadian statistics, it was revealed that fewer than 20% of Canadians do enough exercise [34]. Based on the Centers for Disease Control and Prevention [35], less than 20% of Americans meet the aerobic and muscular activity recommendations. Furthermore, participants had to be overweight or obese (BMI ≥ 25.0), sedentary, healthy, and capable of completing the physical activity program. Studies were included if the sample was comprised of postmenopausal women and men, provided that the findings were reported separately for women. Studies were excluded if the participants had a major medical disorder or condition such as cardiovascular disease, diabetes, or recovering from a stroke.

#### 2.2.3. Types of Interventions

All studies had to contain a physical activity intervention component. Studies that included several groups, diet-only, diet and physical activity, physical activity-only, and the control study arm, were only included if the results of physical activity-only versus the control group were provided. Diet-only interventions were excluded. All types of physical activity interventions such as walking, bicycling, aerobics, stretching, yoga, and resistance training were included while studies with rehabilitation physical activity were excluded.

#### 2.2.4. Types of Outcome Measures

The outcomes included various adiposity, physical capacity, and mental health measures. The adiposity indicators include body weight, total and percentage of fat mass, lean body mass, intra-abdominal and subcutaneous fat, and waist and hip circumference. The physical capacity measures include physical performance, maximal fitness, and pedometer reading. Finally, the mental health outcomes include anxiety, depression, stress, and quality of life. Biochemical outcomes such as level of oxidative stress, C-reactive protein, low- or high-density lipoprotein, and levels of vitamin D were excluded because the literature vastly covers this domain with meta-analyses [36, 37].

### 2.3. Study Selection

**Figure 1:**
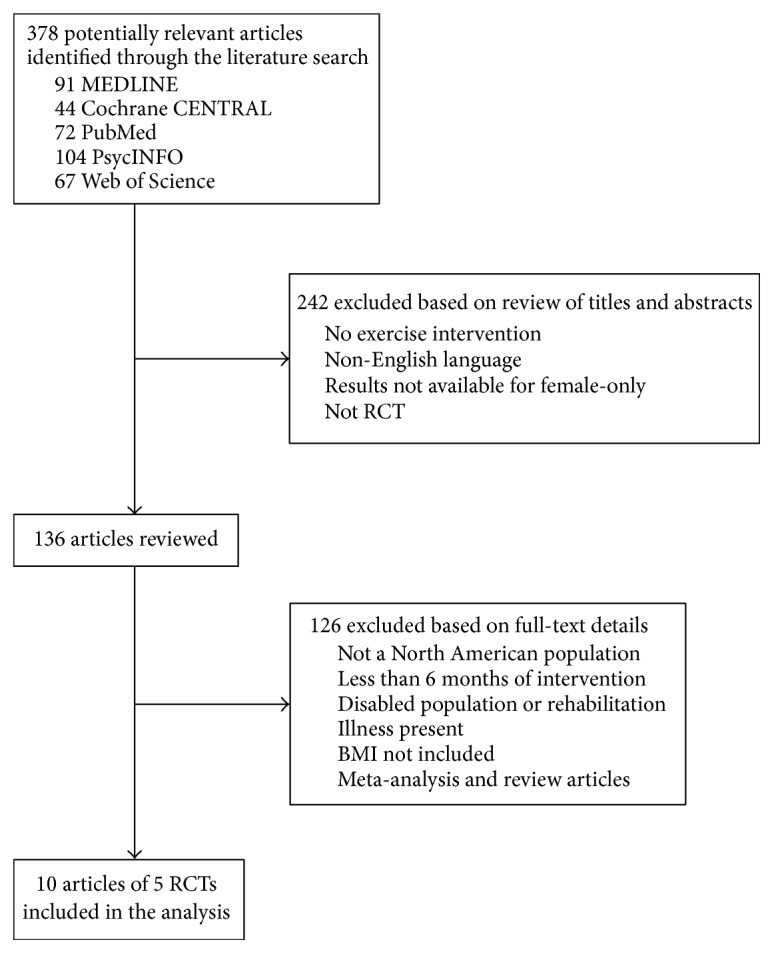
The progressive flow of the study exclusion process.

Initially, titles were reviewed to ascertain the potential fit to the inclusion criteria. If the relevance was doubted during the title review, a subsequent assessment was conducted. The list of potential articles was further shortened by reviewing abstracts and performing detailed evaluations of Sections 2 and 3 of each remaining article (see Figure 1 for the progressive flow of the study exclusion process). Decisions for inclusion were made and verified by one reviewer (AB). If multiple articles were published from the same sample, the article with the most detailed report for each type of physical activity was included and the other reports were only included if they provided new analyses for different outcome variables.

### 2.4. Data Extraction

#### 2.4.1. Risk of Bias

The Cochrane Collaboration tool from the Cochrane Handbook for Systematic Reviews of Interventions [38] was employed to assess the risk of bias for each study. The items in the Cochrane risk of bias assessment include randomization, allocation concealment, blinding of participants and personnel, blinding of outcome assessors, incomplete data, and selective reporting. In addition, the risk of bias for the time of year in which the training took place, trainer qualifications for administration of the intervention, and the suitability of recruitment was assessed using the Cochrane Criteria.

#### 2.4.2. Outcome Data

One researcher (AB) extracted the data for all outcome measures on adiposity, physical capacity, and mental health. Outcome variables were assessed by a wide variety of measures. The adiposity indicators were relatively homogeneous and measured the variable distinctly (i.e., weight, BMI, and circumference, each measuring body size or mass). Similarly, the physical capacity measures distinctly evaluated capability (i.e., maximal fitness, speed, and performance). The mental health outcomes, however, covered an assortment of variables (i.e., stress, anxiety, depression, and quality of life).

#### 2.4.3. Technique Taxonomy

Interventions that are designed to change behavior, such as increasing physical activity among postmenopausal women, are often versatile and incorporate a number of interacting techniques [39]. When strategies are not clearly described or labelled, challenges for replication and application arise [40, 41]. Indeed, current reporting of techniques in published articles is generally poor and frequently omits details making it difficult to analyze and synthesize [42]. According to Michie et al. [43], the lack of such information reduces the reliability and usefulness of the research. To deal with the difficulty, Michie et al. [41, 43, 44] established a comprehensive list of technique classifications based on past behavior change interventions and psychology. The taxonomy is useful for identifying and coding the various methods and techniques applied in any intervention. It provides a system for researchers and clinicians to report interventions in a reliable and universal style. The international behavior change technique taxonomy was applied to the RCTs to identify and summarize the common and/or unique techniques involved. The use of the taxonomy will help describe weight loss program interventions and has the potential to expand weight control study methodologies to improve results, as well as permitting better analyses of multiple studies. Applying the taxonomy to obesity intervention programs among postmenopausal women, according to our knowledge, has not been undertaken. It is an essential segment in the current review paper because experts in intervention design and training are pushing for standardized reports to ease the practices necessary for synthesis in research [45]. To reduce coding biases, two researchers (AB and HSL) extracted the data and coded each intervention technique separately based on Michie's taxonomy system [41]. Any discrepancy in coding was discussed and reviewed until consensus was reached.

### 2.5. Data Analysis

First, the findings from the RCTs were summarized in Table 1. The results for the adiposity indicators, physical capacity measures, and mental health outcomes that were reported in two or more of the trials were subjected to a more thorough observational analysis described in the next section. Finally, the technique taxonomy was applied to each RCT in order to effectively analyze the intervention methods.

## 3. Results

The search identified 378 potential articles and, following a review of the titles and abstracts, only 136 met the inclusion criteria. Examination of Sections 2 and 3 prompted the exclusion of 127 articles. As a result, 9 articles from five RCTs remained that met the inclusion criteria. Three articles were from the Dose Response to Exercise (DREW) study [46–48], two articles were from the Physical Activity for Total Health (PATH) study [49, 50], two articles were from the Nutrition and Exercise for Women (NEW) study [51, 52], one article was from the Women on the Move through Activity and Nutrition (WOMAN) study [53], and one article was from the Alberta Physical Activity and Breast Cancer Prevention (ALPHA) study [54] (see Table 1 for a summary).

### 3.1. Overall Description of Studies

#### 3.1.1. Participants

The studies evaluated a total of 1,669 participants from five different samples. The mean age across all samples was 58.8 (range from 57 to 61 years). The five studies included only female participants. The mean BMI across all studies was 30.5 (range 29.2 to 31.8). Across all five studies, the samples consisted mostly of non-Hispanic white women. In four studies, the majority of the samples were highly educated with at least some college education. In terms of employment, 29%, 50%, and 69.5% of women reported working full time in three of the studies, while in two studies they did not report statistics on employment.

#### 3.1.2. Intervention Location

Most RCTs were conducted in the United States (*n* = 4) and one was conducted in Canada. All studies were carried out in metropolitan centers.

#### 3.1.3. Study Design

All studies were parallel-group RCTs (i.e., each participant was randomly assigned to a study arm, and all the participants in each study arm either received or did not receive the intervention) allowing for comparison of physical activity-only intervention against a control or waitlist group. One intervention [51, 52] had comparisons between several groups including physical activity-only, diet-only, diet and physical activity, and a control group; the methods and results pertaining to diet-only and diet and physical activity groups were excluded in the analysis to focus solely on the efficacy of physical activity-only groups versus the control.

#### 3.1.4. Intervention Duration and Intensity

The modal duration of intervention was 12 months (*n* = 3), ranging from 6 to 48 months. Intervention effects are summarized for end-of-trial comparisons only. Any follow-up prior to the end of the study is not part of the evaluation since the long-term effects are of interest.

Intensity of contact with interventionists varied across intervention groups and ranged from 1 to 4 times a week for the duration of the intervention. One study stopped contact after 24 months due to lack of funding [53]. The participants associated with the program that ceased training were expected to continue to follow the physical activity guide on their own for the remaining 24 months of the program. High-intensity contact interventions were primarily exercise classes or training groups with a specialized trainer such as an exercise psychologist or physiologist.

#### 3.1.5. Description of Intervention Techniques

All of the trials (*n* = 5) had comparable physical activity programs as they all applied some form of aerobics exercise at a moderate-to-vigorous intensity (refer to Table 1 for details). Three studies required physical activity that persisted for 45 minutes for 5 days per week, one study required physical activity 4 days a week with varying durations, and one study did not indicate how many days per week but rather indicated a minimum of 150 minutes per week (see Table 2 for a summary of the means). Each RCT measured moderate-to-vigorous intensity physical activity equivalent to a 3.0 or greater Metabolic Equivalent of Task (MET) level, based on the Compendium of Physical Activities [55]. Less than 3.0 MET refers to light physical activity (such as standing, using a computer, and light walking), 3.0–6.0 MET is moderate physical activity (including heavy cleaning, brisk walking, or light sport), and >6.0 MET aligns with vigorous physical activity (such as hiking, jogging, or shovelling). Each study encouraged the participants to use an assortment of equipment to activate different muscle groups and prevent injury.

### 3.2. Observational Study Results

#### 3.2.1. Risk of Bias

The trials did not show a high risk of bias on any of the items of the Cochrane Collaboration tool nor for the additional biases considered (the season in which the training took place, trainer qualifications for administration of the intervention, and the suitability of recruitment), which suggests that the interpretation of the results should not be affected by the study design and implementation methods.

#### 3.2.2. Effects of Interventions

Please refer to Table 3 for the details of each analysis and reported results.

*Adiposity Outcomes*. Adiposity was measured with various indicators including body weight, BMI, percentage of fat mass, lean body mass, intra-abdominal and subcutaneous fat, and waist circumference. Based on the 9 articles reviewed, participants who were part of a long-term intervention program lasting 6 months or more showed a significant decrease in body weight, total percentage of body fat, BMI, intra-abdominal fat, subcutaneous fat, waist circumference, and hip circumference compared to the control group. There was no significant change in lean body mass. The DREW study [46–48] was the only RCT with various physical activity intervention groups. The 3 physical activity intervention groups differed by exercise dose per week. The study found that, regardless of the dose, a decrease in waist circumference was found compared to the control group and the dose of exercise had an incremental effect on the result. However, there was no significant difference in weight or body fat percentage across the three physical activity intervention groups. Each physical activity dosage group significantly differed in weight from the control group. The NEW study [51] ran secondary analyses to further explore how different doses of exercise impacted adiposity measures. They found that women in the highly active group (≥196 min/week) lost most of the weight by the end of the trial relative to the intermediate active group (154–196 min/week), low active group (<154 min/week), and the control group. The same incremental effects were found for BMI, waist circumference, and percentage of body fat. Even minimal doses of exercise showed a benefit on adiposity indicators compared to no physical activity and weight loss is likely to be greater for those who were engaged in larger doses of exercise.

*Physical Capacity Outcomes.* Physical capacity was measured with parameters such as physical performance, maximal fitness (or VO_2_ max, a measure of endurance) and pedometer reading. All of the RCTs demonstrated improved physical capacity by the end of the trials. Specifically, participants in the physical activity intervention showed improved maximal fitness, lower walk times to complete the 400 meter, and increased pedometer readings compared to the control group. According to the DREW study [46], there was a significant difference in mean maximal fitness response (treadmill testing) for all pairwise comparisons (i.e., the process of comparing groups in pairs to judge which of the two demonstrated greater improvement) per treatment groups (low, moderate, and high dose groups) except the low versus moderate dose groups. This demonstrates that the dose of exercise incrementally improved maximal fitness compared to the control group, and, as expected, the highest dosage produced the largest effect while the low and moderately active groups did not differ from one another. Measures of physical capacity significantly improved with long-term interventions ≥6 months.

*Mental Health Outcomes*. Depression, anxiety, and stress were included in two studies [49, 52] as the mental health outcomes; these constructs were assessed with validated surveys. The Brief Symptom Inventory [56] was used to measure anxiety and depression, and the Perceived Stress Scale [57] was used to measure stress. Overall, the various RCTs showed no significant difference between the physical activity group and the control group on anxiety, depression, and stress. In addition, quality of life was included in three studies [47, 49, 52] and also represented a dimension of mental health. Quality of life was measured with the Short Form Health Survey (SF-36) which includes measures of physical and mental health [58]. Bowen et al. [49] and Imayama et al. [52] both reported nonsignificant results in quality of life at the end of the intervention for the physical activity group and the control group. However, Martin et al. [47] found that treatment groups, specifically the moderate and high dose physical activity groups, indeed showed significant improvements on all subscales of quality of life, with the exception of physical quality of life subscale measuring bodily pain, compared to the control group. This finding suggests that moderate and high doses of physical activity may be required for improving quality of life outcomes among postmenopausal women.

#### 3.2.3. Behavior Change Technique Taxonomy

The results from the behavior change technique taxonomy are displayed in Table 4. To summarize, several techniques were identified and labelled with the corresponding taxonomy in brackets. For instance, interventionists demonstrated how to use the equipment in the training facility (*instructions on how to perform a behavior*/*modelling of behavior*) so that the participants could carry out the physical activity tasks. Daily activity logs were documented by the participants to self-monitor (*self-monitoring of behavior*). The activity logs were submitted weekly for review and follow-up with staff (*others monitoring with awareness*). The trainer evaluated the number of calories to be expended each week and provided the information to the participant during training, or general feedback regarding progress was provided at meetings (*feedback on behavior/social support practical*). The heart rate monitor and/or pedometer was used at each training session to ensure that the intensity of the workout was ideal (*biofeedback/self-monitoring of behavior/others monitoring with awareness*). The facility training was always supervised (*others monitoring with awareness*). In terms of rewards, triannual intervention group activities such as hikes were used to drive motivation (*social reward*) and incentives such as water bottles or monetary compensation were guaranteed when milestones were reached (*material reward/incentive*). Regular individual or group meetings were held to discuss goals (*goal setting behavior/goal setting outcome*). Interventionists also provided feedback on progress toward goals (*feedback on behavior/review behavior goals*) or discussed how to conquer barriers when needed (*problem solving coping planning*). Also individualized phone calls or meetings were conducted to examine, ensure, and promote adherence to the program (*social support general/discrepancy between current behavior and goal standard*). Finally, participants were asked to devote time and effort to the intervention in order to make sure that the controlled conditions were met (*commitment*).

The RCTs included in this review evidently employed a number of behavioral change techniques. Each trial used a very similar collection of techniques. The RCTs employed 17 of the 85 possible behavioral change techniques from the technique taxonomy [41]. As observed, some of the techniques were labelled with 2 or more of the universal codes because some strategies consist of overlapping and highly interacting techniques. Many techniques coincided greatly and often lead to coding one technique with a cluster of codes (see [41] for details on technique clustering).

## 4. Discussion

The current systematic review evaluated the impact of physical activity on adiposity indicators, physical capacity measures, and mental health outcomes among sedentary overweight and obese postmenopausal women. Consistent with previous findings in the general population and adult women [59, 60], adiposity outcomes and physical capacity improved significantly with long-term aerobic interventions compared to a control group. Four out of the five RCTs showed a significant decrease in body weight and percentage of fat mass for those in the intervention group compared to the control group. Two of the RCTs examined BMI during follow-up; BMI significantly decreased compared to the control group. In the studies that examined changes in intra-abdominal fat, subcutaneous fat, and waist circumference, the findings also showed a significant decrease in relation to the control group. Three of the studies looked at lean body mass and found that long-term aerobic physical activity did not have a significant effect. This finding was not surprising because resistance training, which was not employed in any of the trials in this review, is ideal for building lean muscle. For example, Willis et al. [61] demonstrated that overweight and obese adults who participated in an 8-month resistance training program significantly increased in lean body mass compared to the aerobics training group. Conversely, the aerobics training group significantly reduced total body mass and fat mass compared to the resistance training group. In terms of the physical capacity measures, fitness level, as measured by treadmill tests for maximal fitness, relative fitness, and power output, significantly improved among the intervention group compared to the control group. Results of this review, therefore, clearly call for physical activity interventions for postmenopausal women to assist in reduced adiposity and improved physical capacity.

In line with a review conducted by Warburton et al. [62], the results also revealed an incremental relationship between dose of physical activity and change in adiposity indicators and physical capacity measures. As physical activity dose increased, adiposity measures including weight, percentage of body fat, and waist circumference significantly decreased (BMI was found marginally significant). Furthermore, only one study found that, at the end of the 6-month intervention, body weight and percent of body fat did not significantly decrease for any of the physical activity dose groups (low, moderate, and high) compared to the control group. Yet, waist circumference significantly decreased for each dose group compared to the control group and an incremental effect was found. This finding might be due to the fact that the intervention was 6 months in duration and, according to previous research, 6 months or less is relatively short term for changes in body mass and other biochemical responses [63].

Two of the RCTs looked at mental health outcomes including depression, anxiety, and stress. According to these studies, the mental health outcomes did not differ significantly by treatment condition at the end of the 12-month intervention period. The finding does not reflect the results from previous studies with alternative populations [64] (e.g., overweight adults with type II diabetes and adults recovering from stroke). Research has shown that physical activity indeed improves self-reported levels of stress, anxiety, and depression in these populations [65–67]. Yet, this is not what we found. Imayama et al. [52] proposed that the participant's preference for type of physical activity could have affected the results because if walking on a treadmill was not their preferred activity, it may have impacted the self-report responses on mental health outcomes. Furthermore, participants in the trials reported relatively low levels of depression and anxiety at baseline which may have caused a “basement effect” (also known as the floor effect). This effect is often established in research when participants report low levels on a measure at baseline and also at follow-up. Consequently, it becomes difficult, if not impossible, to find significant improvement on the measure (in this case depression and anxiety) since the subjects initially reported little to no symptoms. Finally, mental well-being is typically influenced by a number of factors and physical activity may not necessarily improve every aspect of an individual's life to facilitate a change in depression, anxiety, and stress scores. Rather, physical activity may help improve more immediate consequences such as mood or affect, and future work should explore this realm of mental health. Given that there is a limited number of studies that looked at mental health outcomes, future research should continue to investigate the effects of physical activity on stress, anxiety, and depression since there is not enough evidence to reliably infer and draw conclusions.

Interestingly, one study found that the control group of stretchers significantly improved on depression scores [49]. This finding provides evidence for the value of stretching and breathing techniques in physical activity programs. Previous research explored the impact of yoga and stretching on state and trait anxiety [67] and on depression [68]. The literature demonstrates that stretching has a positive impact on various mental health outcomes. Improvement on mental health outcomes may have been significant for the intervention group if this type of activity was incorporated in the training sessions. On the other hand, if the control group did not incorporate an intervention that is typically used to treat symptoms of depression and anxiety, group differences may have been observed on the mental health outcomes.

Quality of life was also examined and considered a dimension of mental health. In line with a recent systematic review and meta-analysis [69], weight loss was not significantly associated with overall quality of life. Specifically, Warkentin et al. [69] found that weight loss might be associated with modest improvements in the physical aspect of quality of life,but not the mental health aspect. Similarly, McCarroll et al. [70] found that adult endometrial cancer survivors, who were assigned to a weight loss intervention to reduce the risk of cardiovascular disease, demonstrated that quality of life had no correlation to weight loss. However, a recent study by Rothberg et al. [71] found that a greater reduction in BMI after the physical activity intervention significantly improved quality of life measures and individual differences appeared to influence the strength of the association. Participants with a lower baseline BMI, greater decrease in BMI from baseline to follow-up, and poorer baseline quality of life score demonstrated greater improvements in quality of life at follow-up.

The current review tends to share the contradictory nature of the findings from previous work. Two of the studies [49, 52] reported nonsignificant results on quality of life by treatment condition and one study [47] found remarkable results. Namely, Martin et al. [47] found that the dose of physical activity had an effect on self-reported quality of life. More explicitly, the social quality of life subscales (social functioning and role limitation) demonstrated significant improvement among moderate and high physical activity dose groups compared to the control group. The remaining two subscales for social quality of life (vitality and mental health) demonstrated a significant improvement for all physical activity groups (low, moderate, and high) compared to the control group. In terms of the physical quality of life subscales, both physical functioning and role limitations improved significantly among moderate and high physical activity dose groups compared to the control group. The physical quality of life subscale measuring general health perceptions significantly improved for all doses of physical activity groups compared to the control group. Lastly, the physical quality of life subscale measuring bodily pain did not show significant results for any groups. These findings may suggest that higher doses of physical activity are necessary for improving quality of life outcomes. Future research is required to understand such inconsistencies.

The Canadian Physical Activity Guidelines for Adults suggest that 150 minutes of physical activity per week is the minimum requirement [72]. All five RCTs met this criterion. The results demonstrate that the requirement of 150 minutes per week of physical activity is a suitable guideline for the samples of postmenopausal women included in this review. In general, the findings indicate plausible efficacy of long-term physical activity interventions. To further strengthen the evidence base, future research should include a meta-analysis of the effectiveness of physical activity on various outcomes, focusing particularly on those that have implications for mental health. Such prospective research will help develop a better understanding of physical activity interventions among sedentary overweight and obese postmenopausal women.

### 4.1. Classification of Behavior Change Techniques

The classification of strategies from the behavior change technique taxonomy [41] was applied to standardize the intervention content across the RCTs and identify the different assortment and levels of intervention techniques. The findings revealed that the techniques were largely focused on two levels: the individual (intrapersonal) and the social aspect (interpersonal) of behavior change. The techniques used to change the subject's behavior included* intrapersonal* strategies to help educate and increase knowledge, teach new skills, and develop competence, whereas the* interpersonal* techniques were employed to help promote social support and encourage adherence to the program. Indeed, the two levels of techniques are crucial in improving physical activity behavior among postmenopausal women as they evidently helped produce positive effects on adiposity and physical capacity.

Other techniques may have been applied in the interventions, but the content was either not transparent or the description was too brief. Michie et al. [43] have emphasized the importance of reporting details and the taxonomy is simply a system to help accurately document the intervention content. Nevertheless, similarities and differences across the interventions were uncovered based on the taxonomy analysis. The interventions centered their techniques on instruction, education, modelling, feedback, and rewards. Techniques such as manipulating or restructuring the environment (physical or social) or targeting emotions were not used in any of the interventions.

The Social Ecological Model of Health Behavior developed by McLeroy et al. [73] is a behavior change framework which emphasizes that a variety of sources influence specific health behaviors and they do so by interacting with one another. The framework has been used to understand and identify the relevant sources for behavior-specific interventions such as smoking cessation [74, 75] and healthy eating [76]. The model includes five main sources or levels of influence on health behaviors: the intrapersonal (characteristics of the individual including skills, attitudes, and developmental history), interpersonal (social networks and support systems), organizational (institutional and organizational cultures, rules, and regulations for operation), community (relationships among organizations, institutions, and other networks), and public policy (local, provincial, and national laws). Ample research proposes that multilevel interventions that incorporate various levels from the Social Ecological Model are ideal for improving large-scale or population-wide health problems such as obesity [77].

Multilevel models lead to more substantial and sustained changes in health behaviors compared to single-level or dual-level interventions because they provide opportunity for maximal behavior change [78, 79]. According to the World Health Organization [1], health behaviors improve when environments and policies support healthy choices and individuals are motivated and educated to make those choices. Educating people to make healthy choices when environments are not supportive may not be effective in making behavioral change because the environment provides norms, rules, and resources to behave in a particular way [73, 80].

The environmental approach seems to be growing in physical activity intervention research. For example, a study by Carlson et al. [81] found that environmental features and psychosocial measures of self-efficacy and social support are associated with moderate-to-vigorous physical activity among older adults. Specifically, those who reported a high level of support to exercise from family and friends had on average 56 minutes of more physical activity per week compared to those who reported low social support. As well, environments that were reported as having pleasing aesthetics were associated with increased physical activity by roughly 30 minutes per week compared to areas reported as having undesirable aesthetics. Negative perceptions of safety, traffic, and distances from exercise facilities were found to reduce physical activity by about 18 minutes per week [82]. Chaudhury et al. [82] found that environmental aesthetics such as smooth walking surfaces (trails and parks around the community), plenty of benches for comfort and breaks, safety and security, and peer support (partners for activities) were found to be associated with increased physical activity regardless of population density in the neighbourhood. Evidently, social and environmental aspects should be considered by interventionists. In order to truly drive physical activity intervention research forward, environmental levels should be integrated in research.

Several countries have reported major health improvements from implementing a multilevel approach focusing on people, policy, and environment such as Brazil [83] and Costa Rica [84]. Supporting a person-environment approach seems to be the gold standard in intervention research. To advance physical activity intervention research and build from the current systematic review, these multilevel approaches could serve as models for testing the effectiveness of physical activity interventions in North American postmenopausal populations.

### 4.2. Strengths and Limitations

The main strength of this review is the comprehensive and meticulous search of five databases and the synthesis of available evidence from RCTs of physical activity interventions among sedentary postmenopausal women in Canada and the United States. The findings, however, may only generalize to the contexts in which the studies have been conducted. Researches in different countries, cultural settings, and postmenopausal women with serious health conditions require a separate investigation. Different clinical populations tend to have specific characteristics that require tailored interventions especially if the subjects are unable to walk themselves to a facility for training. Some clinical populations may suffer from fatigue or have priority treatment groups like patients battling cancer. A systematic review by Knols et al. [85] examined the benefits of physical activity in cancer patients. The review found statistically significant results favouring even small doses of walking and strengthening exercises (free weights and isokinetic machines) on body fat, lean body mass, bone mineral density, muscle strength, walking distance, and self-reported outcomes including fatigue, psychological well-being, mood, and quality of life. The findings revealed that low levels of physical activity (whether carried out in a rehabilitation program or at home) are effective in improving the physical and psychosocial functioning of patients with breast cancer both during medical treatment and after it. Zanuso et al. [86] also suggested that intervention research among some clinical populations should focus on not only the effect of aerobic exercise itself, but also the effect of exercise intensity. Prospective research should therefore investigate postmenopausal women with health conditions as they may require different interventions and evaluations.

A second strength is that the adiposity indicators and physical capacity tests were objectively measured and recorded with sophisticated instruments such as a scale to measure body weight, ultrasounds to measure fat mass, intra-abdominal fat, and subcutaneous fat, a treadmill test to measure fitness, and a pedometer to measure steps.

A limitation is that the mental health outcomes were measured with self-report methods. Issues and restrictions with self-reported outcomes have been flagged in the literature [87] and may have had an impact on the results. Additionally, very few trials (*n* = 2) examined the impact of physical activity on diverse mental health outcomes among postmenopausal women and future research should explore this avenue to understand the implications of physical activity on mental well-being.

Sisson et al. [48] noted that, after running individual-level analyses, a fair amount of individual variability in response to maximal fitness became apparent. They found that the volume of training and age played a role on maximal fitness results. Stratified sampling by BMI, age, or ethnic group was also not carried out for any of the outcome measures. Individual variability such as differences in cultural or ethnic groups may exist, but this information was not included in the original RCTs. Sublevel analyses ought to be carried out in future work.

The methodological quality of some of the studies demonstrated room for improvement as judged by the Cochrane Collaboration tool for assessing risk of bias [38]. Many of the RCTs did not report methods for blinding of participants, personnel, and outcome assessors. Half of the studies failed to report the process for concealing the allocation of participants and how the allocation was masked throughout the trial period. The missing content was not judged to be a high risk; however, future reports should improve the coverage of this content. Likewise, the particular behavioral change techniques employed in the interventions to support and increase physical activity were described with little details. The consistent lack of information tends to hinder the reliability and usefulness of intervention studies because such details are required for replication and application elsewhere [43]. The missing information also creates a challenge for reliably translating and interpreting the various techniques in secondary research such as systematic reviews [41]. Though the potentially omitted information is a limitation, this expected circumstance provides a learning opportunity for future intervention reports where all relevant details should be provided.

Lastly, the inclusion criterion to select and investigate RCTs presents another restraint. Although the review of RCTs is a reliable means to establish efficacy, the effectiveness of physical activity interventions among postmenopausal women was not examined. Consequently, it was not possible to study the effects of the environment since the environments were highly controlled during training. Based on the notion that environment and policy are key in maximal behavior change and maintenance, prospective work should focus on investigating the effectiveness of such multilevel intervention models.

## 5. Conclusion

The current systematic review evaluated the efficacy of physical activity interventions on adiposity indicators, physical capacity measures, and the mental health outcomes of stress, anxiety, and depression among sedentary overweight/obese postmenopausal women. The other goal of the review was to identify the various levels of behavioral change techniques that were employed in the interventions to determine the common techniques and guide prospective research and application in alternative settings.

The findings showed that addressing the intrapersonal and interpersonal levels of behavior change improved physical activity levels which led to a number of positive outcomes on adiposity indicators and physical capacity measures. Future research should explore physical activity interventions among postmenopausal women in terms of a multilevel model going beyond the combination of intrapersonal and interpersonal sources. Although the literature does not comprise many studies on physical activity interventions among postmenopausal women and certainly no studies that follow a multilevel approach, this is recommended as the direction for future work. Given that multilevel models take a combination of both individual- and environmental-level intervention techniques to maximize change in health behaviors among populations, this framework would seemingly work well for physical activity programs geared toward postmenopausal populations. In turn, the issue of overweight and obesity among postmenopausal women may, in due course, subside.

## Figures and Tables

**Table 1:** Study descriptions and summary of results: randomized controlled trials in North American populations.

Study acronym and source	Recruitment	Population	Duration, months	Number of subjects	Intervention and control description	Intervention details	Outcome variables	Results
*PATH*								
Bowen et al., 2006 [49]	Mass mailings and media placements (American)	50–75 years, otherwise healthy, sedentary, BMI ≥ 25, women after menopause	12	173	PA intervention: 45 min of moderate-intensity aerobic exercise 5 days/week (incl. walking, bicycling, and aerobics). Participants attended at least three mandatory training sessions held at the facility supervised by an exercise psychologist for months 1–3. The remaining two sessions were to be done at home. In months 4–12, participants had to attend at least one session/week at the facility and exercise at home for the remaining days. Some resistance training was recommended. Control: 45 min stretching session once/week for the year.	Daily activity log to record type of exercise, duration, peak heart rate, and rating of perceived exertion for all sport and recreational activities. Questionnaires at baseline and 12 months, and Polar HR monitors.	Anxiety and depression: subscales from the Brief Symptom Inventory (BSI). Perceived stress scale: measures stress response to events from the last month.	No significant difference in mental health at 12 months between groups. No significant difference was found for anxiety and depression between groups at 12 months. Perceived stress did not differ significantly between groups at 12 months. Yet, perceived stress improved significantly among the control group between baseline and 12 months.
Campbell et al., 2010 [50]							Maximal O_2_ uptake (cardiorespiratory fitness) measured with treadmill test. Anthropometry: weight, waist and hip circumference, total body fat and percentage of body fat, and lean body mass. Intra-abdominal and subcutaneous fat measured by medical scan	Exercisers significantly increased in maximal fitness compared to control group (*p* = 0.0001). At the end of 12 months, exercisers decreased body weight (*p* = 0.007), body fat percentage (*p* = 0.001), intra-abdominal fat (*p* = 0.045), and subcutaneous fat in exercisers (*p* = 0.003) compared to the control group. Waist circumference significantly decreased among exercisers compared to the control group (*p* < 0.05). No significant difference between exercisers and the control group in change in lean body mass after 12 months.

*NEW*								
Foster-Schubert et al., 2012 [51]	Mass mailing campaigns, media publicity, or community outreach prompted calls (American)	50–75 years, otherwise healthy, sedentary, BMI ≥ 25, women after menopause	12	204 (PA-only and control group)	PA intervention: 45 min of moderate-intensity aerobic exercise 5 days/week (walking/hiking, bicycling, and aerobics). Participants attended at least three mandatory training sessions held at the facility and supervised by an exercise psychologist. These sessions included use of treadmill, stationary bike, and other aerobic machines. The remaining two sessions were to be done at home. Some resistance training was encouraged to prevent injury. Within the first 7 weeks, PA sessions were 15 minutes at 60–70% observed maximal heart rate and gradually worked up to 70–85% where they continued for the duration of the study. Control: no change to habits or lifestyle.	Activity log to record type of exercise, duration, peak heart rate, and rating of perceived exertion. Use of Polar HR monitors and pedometer.	Anthropometry: body weight waist circumference Body composition: percentage body fat and lean body mass. Maximal fitness measured with treadmill test. Pedometer reading measured steps walked/week.	Among exercisers, body weight decreased significantly (*p* = 0.034) compared to the control group. Waist circumference and percentage of body fat decreased significantly among exercisers compared to the control group (*p* = 0.001 and *p* < 0.0001, resp.). Lean body mass did not differ significantly across the two groups. Exercisers were classified in 1 of 3 categories for further analysis: highly active (≥196 min/week physical activity), intermediate active (154–196 min/week), and low active (<154 min/week). Women in the highly active group lost the most weight (*p* < 0.0001) relative to the other groups and the controls. The same trend was found for BMI, waist circumference, percentage of body fat, maximal fitness, and change in pedometer steps/week. Hence, highly active exercisers demonstrated the largest change compared to all other exercise groups.
Imayama et al., 2011 [52]	Mass mailings and media placements (American)						Maximal fitness measured with treadmill test. Depression and anxiety: Brief Symptom Inventory-18. Stress: Perceived Stress Scale.	Maximal fitness increased significantly in exercisers compared to controls after the 12 months (*p* < 0.001). Perceived stress, anxiety, and depression scores did not significantly change among exercisers compared to controls. Social support was nonsignificant between groups after the 12 months.

*DREW*								
Church et al., 2007 [88]	Ads in newspaper, radio, television, community events, and mass mail (American)	45–75 years, otherwise healthy, sedentary, BMI 25–43, women after menopause	6	464	PA intervention group 1: participants attended 4 training sessions/week which included cycling and treadmill expending 4 kilocalories per kilogram of body weight per week. Energy expenditure level is about 50%. PA intervention group 2: participants attended 4 training sessions/week which included cycling and treadmill expending 8 kcal per kg/week. Energy expenditure level is about 100% PA intervention group 3: participants attended 4 training sessions/week which included cycling and treadmill expending 12 kcal per kg/week. Energy expenditure level is about 150% All intervention groups experienced moderate-to-vigorous intensity activity. Groups differed by amount of time spent exercising. Control group: asked to maintain current lifestyle	Trainers documented progress. HR monitors and pedometers were used.	Pedometer measures steps/day. Fitness measures: peak maximal fitness, peak relative fitness per min, and maximal power output, measured with treadmill test. Weight, body fat percentage, and waist circumference	After the 6 months, there were no significant differences on steps per day across all the groups. For all 3 fitness measures, each exercise dose had a significantly higher impact compared to the control group (*p* < 0.001 for each group). There was no significant difference in weight or body fat percentage. Waist circumference was significantly smaller in all 3 exercise groups compared to the control group (*p* < 0.05 for each). The dose of exercise has an incremental effect on the results.
Martin et al., 2009 [47]	Ads in local community and team recruitment in minorities communities (American)			430			Quality of life (QOL): Medical Outcomes Study Short Form Health Survey (SF-36) that measures physical (4 subscales) and mental (4 subscales) QOL body weight. Maximal O_2_ uptake (cardiorespiratory fitness) measured with cycling test.	A significant dose-response effect of exercise on QOL was found for 3 of 4 subscales of physical health (i.e., physical functioning, role limitations due to physical problems, and general health perception) and 4 of 4 scales of mental health (role limitations due to emotional problems, social functioning, vitality, and mental health). Post hoc analysis demonstrated that the 12 kcal per kg/week group significantly improved QOL compared to the controls. The 4 kcal per kg/week group significantly improved general health, vitality, and mental health. Body weight and maximal fitness had no effect on QoL outcomes.
Sisson et al., 2009 [48]	Ads in local community and team recruitment in community (American)			464			Maximal O_2_ uptake (cardiorespiratory fitness) measured with cycling test.	A significant difference in maximal aerobic fitness was found for all pairwise treatment group comparisons except 4 kcal per kg/week versus 8 kcal per kg/week.

*WOMAN*								
Gabriel et al., 2011 [53]	Direct mailing from selected ZIP codes (American)	52–62 years, otherwise healthy, sedentary, BMI 25–39.9, women after menopause	48	508	Intervention: 150 minutes per week of moderate-intensity activity similar to brisk walking. Participant contact was extensive and included 40 group visits in first year, 12 monthly visits in years 2 and 3, and no visits in year 4. Visits included a 400-meter walk as fast as possible and physical activity follow-up questionnaire Control: health education group received 6 lectures in year 1 and then quarterly thereafter but was not instructed to change lifestyle.	Evaluated at 6 months and then annually using 400-meter walk test and MAQ interview-administered questionnaire by specialists. Daily diary was used to log PA every day and monitor adherence.	400-meter walk: 10 laps along a hallway with cones set 20 m apart at a pace that could be maintained the whole duration. Leisure time physical activity (LTPA): measured with subscale of Modifiable Activity Questionnaire (MAQ) which assessed leisure activity over the year Body composition: measure with weight, BMI, waist circumference, and body fat mass.	The exercise group had significantly lower 400-meter walk times compared to the control group (*p* < 0.04). While the exercisers walk time decreased, the control group had no significant change in walk times after the 48 months. Exercisers also had significantly decreased body weight, BMI, waist circumference, and trunk fat mass compared to the control group (*p* < 0.04). For the exercise group, increased LTPA and decreased body weight, BMI, waist circumference, and whole body fat mass were significantly associated with reductions in 400 m walk time from baseline to 48 months (all *p* < 0.01).

*ALPHA*								
Friedenreich et al., 2011 [54]	Targeted mailings, posters, brochures, and media campaigns (Canadian)	50–74 years, otherwise healthy, sedentary, BMI 22–40, women after menopause	12	320	Intervention: participants were instructed to complete 45 min of moderate-to-vigorous intensity aerobic exercise 5 days/week. Within the first 3 months, subjects gradually worked up to 70–80% of their heart rate reserve and then remained at 70–80%. Three mandatory sessions/week were held at the facility with specialized trainers. The remaining 2 sessions were home-based. A warm-up and cool-down of 5–10 minutes were encouraged and carried out at the facility training sessions. Control: asked to maintain current lifestyle.	Daily exercise log and Polar HR monitors.	Maximal fitness was measured with a treadmill test. Energy intake and adiposity measured with intra-abdominal and subcutaneous fat via medical scan. Weight, BMI, waist and hip circumference, body fat, percentage of body fat, and lean body mass were measured using X-ray scans.	Maximal fitness increased significantly more in exercisers compared to controls (*p* < 0.001). Mean energy intake decreased among controls relative to exercisers (*p* = 0.01). The mean decrease in adiposity between baseline and 12 months was significantly greater in exercisers compared to controls (*p* < 0.001). Weight, BMI, waist circumference, hip circumference, percentage of body fat, and abdominal fat area all significantly decreased among exercisers compared to the control group. Lean body mass was not significantly different among exercisers and the control group (*p* = 0.564). Lean body mass did not change for exercisers and decreased slightly for the control group.

**Table 2:** Physical activity descriptions and means.

Study	Aerobics activity	Average minutes of physical activity per week	Average heart rate
PATH	Cycling, walking, aerobics, and some resistance training	171.3	60–70
NEW	Cycling, walking, and aerobics	225	80
ALPHA	Cycling, walking, and aerobics	178.5	62.2
DREW			
Low dose	Cycling and walking	72.2	55.1
Moderate dose	Cycling and walking	135.8	76.2
High dose	Cycling and walking	191.7	80.7
WOMAN	Fast walking	150	—

**Table 3:** Details of the analyses and results per RCT.

Intervention	Authors	Type of analysis	Outcome variables	Results
PATH	Bowen et al., 2006 [49]	Between-group analyses	Perceived stress	*No significant difference *
			Anxiety	*No significant difference*
			Depression	*No significant difference*
			General mental health	*No significant difference*
			Physical functioning	Significantly improved among intervention group compared to control group
			Social support: emotional	*No significant difference*
			Social support: affection	Significantly higher for the control group compared to the exercisers
			Social support: tangible	*No significant difference*
			Social support: overall	*No significant difference*
		
		Within-group analyses	Perceived stress	*No significant difference*
			Anxiety	*No significant difference*
			Depression	(i) No significant difference between baseline and 12-month follow-up for intervention group (ii) Control group of stretchers significantly improved on depression after 12 months
			General mental health	(i) Significantly improved between baseline and 12-month follow-up for intervention group (ii) No significant difference for participants in the control group
			Physical functioning	(i) No significant difference between baseline and 12-month follow-up for intervention group (ii) Control group of stretchers significantly improved on physical functioning after 12 months
			Social support: emotional	*No significant difference*
			Social support: affection	(i) No significant difference between baseline and 12-month follow-up for intervention group (ii) Control group of stretchers significantly improved on affection subscale after 12 months
			Social support: tangible	*No significant difference*
			Social support: overall	*No significant difference*
			
	Campbell et al., 2010 [50]	Between-group analyses	VO_2_ max	Significantly increased by 13.6% compared to the 0.2% increase in the control group
			Body weight	Significantly decreased by 1.3 kg compared to the 0.3 kg decrease in the control group
			Percent of body fat	Significantly decreased by 1.4% compared to the 0.1% decrease in the control group
			Intra-abdominal fat	Significantly decreased compared to the control group
			Subcutaneous fat	Significantly decreased compared to the control group
			Waist circumference	Significantly decreased compared to the control group
			Lean body mass	*No significant difference*

NEW	Foster-Schubert et al., 2012 [51]	Between-group analyses	Pedometer steps	Significantly increased by 42% compared to the control group
			VO_2_ max	Significantly increased by 9% compared to the control group
			Body weight	Significantly decreased by 2.0 kg compared to the 0.7 kg decrease in the control group
			Waist circumference	Significantly decreased by 2.0 cm compared to the 0.9 cm decrease in the control group
			Percent of body fat	Significantly decreased by 1.6% compared to the control group
			Lean mass	*No significant difference*
		
		Within-group analyses stratified by dose of physical activity: <154 min/wk, 154–196 min/wk, and ≥196 min/wk	Body weight	Significantly decreased for only those who did ≥196 min/wk of physical activity
			BMI	Significantly decreased for only those who did ≥196 min/wk of physical activity Marginally significant decreased for those who did 154–196 min/wk of physical activity
			Waist circumference	Significantly decreased for those who did 154–196 and ≥196 min/wk of physical activity
			Percent of body fat	Significantly decreased for those who did 154–196 and ≥196 min/wk of physical activity
			
	Imayama et al., 2011 [52]	Between-group analyses	Quality of life: physical functioning	*No significant difference*
			Quality of life: role physical	*No significant difference*
			Quality of life: vitality	*No significant difference*
			Quality of life: mental health	*No significant difference*
			Perceived stress	*No significant difference*
			Anxiety	*No significant difference*
			Depression	*No significant difference*
			Social support	*No significant difference*
		
		Within-group analyses	Perceived stress	*No significant difference*
			Anxiety	*No significant difference*
			Depression	*No significant difference*
			Social support	*No significant difference*

DREW	Church et al., 2007 [88]	Between-group analyses	Body weight	*No significant difference*
			Percent of body fat	*No significant difference*
			Waist circumference	Significantly decreased in all intervention groups compared to the control group
			Peak power output	Significantly increased in all intervention groups (7.6%, 10.7%, and 12.9%, resp.)
			Peak maximal fitness	Significantly increased in all intervention groups (4.2%, 6.0%, and 8.2%, resp.)
			Peak relative fitness	Significantly increased in all intervention groups (4.7%, 7.0%, and 8.5%, resp.)
		
	Martin et al., 2009 [47]	Between-group analyses	Physical quality of life: physical functioning	Moderate and high doses of physical activity significantly improved compared to control group
			Physical quality of life: role limitations	Moderate and high doses of physical activity significantly improved compared to control group
			Physical quality of life: bodily pain	*No significant difference*
			Physical quality of life: general health perceptions	All doses of intervention groups significantly improved compared to the control group
			Social quality of life: social functioning	Moderate and high doses of physical activity significantly improved compared to control group
			Social quality of life: role limitation	Moderate and high doses of physical activity significantly improved compared to control group
			Social quality of life: vitality	All doses of intervention groups significantly improved compared to the control group
			Social quality of life: mental health	All doses of intervention groups significantly improved compared to the control group
	Sisson et al., 2009 [48]	Between-group analysis	VO_2_ max	Significantly increased per pairwise comparison except the low dose versus moderate dose of physical activity

WOMAN	Gabriel et al., 2011 [53]	Between-group analyses	Body weight	Significantly decreased compared to the control group
			BMI	Significantly decreased compared to the control group
			Waist circumference	Significantly decreased compared to the control group
			Fat mass	Significantly decreased compared to the control group
			400-meter walk time	Significantly decreased compared to the control group
	
		Within-group analyses	Body weight	Significantly decreased between baseline and 12-month follow-up
			BMI	Significantly decreased between baseline and 12-month follow-up
			Waist circumference	Significantly decreased between baseline and 12-month follow-up
			Body fat mass	Significantly decreased between baseline and 12-month follow-up
			400-meter walk time	(i) Marginally significant decrease for intervention participants between baseline and 48-month follow-up
				(ii) No change for control group participants between baseline and 48 months

ALPHA	Friedenreich et al., 2011 [54]	Between-group analyses	Body weight	Significantly decreased by 2.3 kg compared to the 0.5 kg decrease in the control group
			Body fat mass	Significantly decreased by 2.4 kg compared to the 0.4 kg decrease in the control group
			Abdominal fat	Significantly decreased by 16.5 cm^2^ compared to the 9.6 cm^2^ decrease in the control group
			Waist circumference	Significantly decreased by 2.2 cm compared to the 0.1 cm decrease in the control group
			Lean body mass	*No significant difference*
			VO_2_ max	Significantly increased by 14.2% compared to the 2.6% increase in the control group

**Table 4:** The behavior change technique taxonomy applied to the selected randomized controlled trials.

Taxonomy	Behavioral change technique applied in intervention	RCT
Instructions on how to perform a behavior	Demonstrate equipment	PATH, NEW, and ALPHA

Modelling of behavior	Demonstrate equipment	PATH, NEW, and ALPHA

Others monitoring the behavior with awareness	Review activity logs	PATH, NEW, and ALPHA
	Heart rate monitor	PATH, NEW, DREW, and ALPHA
	Pedometer reading	DREW and WOMAN
	Supervised training	PATH, NEW, DREW, and ALPHA

Self-monitoring of behavior	Activity log	PATH, NEW, and ALPHA
	Pedometer reading	DREW and WOMAN
	Heart rate monitor	PATH, NEW, DREW, and ALPHA

Feedback on behavior	follow-up on activity logs	PATH, NEW, and ALPHA
	Provide information about calories to burn	DREW
	Information sessions	WOMAN
	Feedback on progress	WOMAN

Biofeedback	Heart rate monitor	PATH, NEW, DREW, and ALPHA
	Pedometer reading	DREW and WOMAN

Social rewards	Triannual activities like hiking	PATH

Material rewards	Water bottles	PATH
	Money	DREW

Other incentives	Not identified	ALPHA

Goal setting behavior	Individual meetings to discuss goals	PATH and ALPHA
	Group meetings to discuss goals	NEW

Goal setting outcome	Individual meetings to discuss goals	PATH and ALPHA
	Group meetings to discuss goals	NEW
	Target to meet dose of physical activity	DREW

Reviewing behavior goals	Feedback on progress toward goals	PATH, NEW, and ALPHA

Discrepancy between current behavior and goal standard	Phone meetings to ensure adherence	PATH and ALPHA
	Individual face-to-face meetings to ensure adherence	PATH and ALPHA
	Group meetings to ensure adherence	NEW

Social support practical	Follow-up regarding activity logs	PATH, NEW, and ALPHA
	Provide information about calories to burn	DREW
	General feedback on progress	PATH, NEW, DREW, WOMAN, and ALPHA

Social support general	Phone meetings	PATH and ALPHA
	Individual face-to-face meetings	PATH and ALPHA
	Group meetings	NEW

Problem solving coping planning	Discuss how to conquer barriers	NEW

Commitment	Asked to devote time and effort for duration of intervention	PATH, NEW, DREW, WOMAN, and ALPHA
